# HTLV-1 Tax directly modulates expression of the CDK6 and RNASET2 genes

**DOI:** 10.1186/1742-4690-8-S1-A153

**Published:** 2011-06-06

**Authors:** Nicholas Polakowski, Hongjin Han, Isabelle Lemasson

**Affiliations:** 1 Department of Microbiology and Immunology, Brody School of Medicine, East Carolina University, Greenville, NC, 27834, USA

## 

The viral protein Tax utilizes interactions with many transcriptional regulatory proteins to modulate cellular gene expression and to activate transcription of the Human T-cell Leukemia Virus type 1(HTLV-1) provirus. In some cases it is possible that effects of Tax on cellular genes involve direct association of the viral protein with the promoter of the gene. Using a chromatin immunoprecipitation assay in conjunction with microarray and subsequent real-time PCR analysis, we identified and confirmed the association of Tax with promoters for the CDK6 and RNASET2 genes. These genes are of interest because they play roles in cellular proliferation and cell survival, respectively, which are processes affected by Tax. Cdk6 is a cyclin-dependent kinase most recently implicated in sustaining cells in an undifferentiated state. RNaseT2 is a ribonuclease that likely functions as a tumor suppressor in human cells. Interestingly, ectopic cellular expression of Tax led to increased Cdk6 mRNA and protein levels, while reducing those levels for RNase T2, suggesting that Tax utilizes distinct mechanisms to regulate expression of each gene. We have mapped the Tax binding sites to DNA regions centered at -468 and 618 relative to the RNA start site in the CDK6 and RNASET2 promoters, respectively. Studies are ongoing to understand how Tax regulates the expression of each gene.

